# Role of Qualitative and Quantitative Indocyanine Green Angiography to Assess Mastectomy Skin Flaps Perfusion in Nipple/Skin-Sparing and Skin-Reducing Mastectomies with Implant-Based Breast Reconstruction

**DOI:** 10.1155/2022/5142100

**Published:** 2022-03-31

**Authors:** Manuela Mastronardi, Stefano Fracon, Serena Scomersi, Margherita Fezzi, Marina Bortul

**Affiliations:** ^1^Division of General Surgery, Department of Medical and Surgical Sciences, Cattinara University Hospital, Strada di Fiume 447, Trieste 34149, Italy; ^2^Breast Unit, Cattinara University Hospital, Strada di Fiume 447, Trieste 34149, Italy

## Abstract

**Methods:**

Consecutive women scheduled for nipple/skin-sparing/skin-reducing mastectomy between May 2020 and April 2021 were prospectively enrolled. Patients were divided into Group 1 in the absence of superficial and full-thickness necrosis (SN; FTN) and Group 2 in the presence of both. T1 (time between ICG injection and the initial perfusion of the least perfused MSF area), ICG-Q1, and ICG-Q% (absolute and relative perfusion values of the least vascularized area) were collected.

**Results:**

38 breasts were considered. FTN was reported in 4 breasts (10.5%) and SN in 3 (7.9%). The two groups statistically differ in T1 (Group2 > Group1) and ICG-Q% (Group1 > Group2) (*p* < 0.05). T1 could statistically predict ICG-Q1 and ICG-Q%. Both quantitative values have a sensitivity of 57% and a NPV of 89%; ICG-Q% shows higher specificity (81% vs 77%) and PPV (40% vs 36%).

**Conclusions:**

Quantitative ICG angiography may additionally reduce MSF necrosis. Moreover, longer T1 may indicate possible postoperative necrosis. Considering these factors, intraoperative changes of reconstructive strategy could be adopted to reduce reconstructive failure.

## 1. Introduction

In the past few years, breast cancer surgical treatment has become more and more conservative. Nevertheless, in 25–30% of cases, a mastectomy is demanded. Nowadays, breast surgeons try to spare skin and nipple-areola complex (NAC), when possible, and to offer an immediate breast reconstruction (IBR) [1–3]. Compared to delayed breast reconstruction, IBR has become more and more relevant, allowing a better outcome in terms of patients' satisfaction level [2–6]. Mastectomy techniques are so improved to allow the skin and NAC preservation with the same oncological safety of simple mastectomies, where skin and NAC are removed [7, 8]. Moreover, the postoperative complication rate of skin and NAC-sparing mastectomies is around 20–30%, needing a medical or surgical treatment in 10–12% of cases [9]. The most frequent complications are mastectomy skin flaps (MSF) necrosis, surgical site infection (SSI), the presence of a seroma and/or hematoma, and the implant removal, determining a reconstructive failure [10]. In particular, MSF necrosis has an incidence of 10–20%, needing to take-off the implant in 8–18% of cases, and a delay in systemic therapy beginning, when required [10, 11]. This clearly explains the importance of the intraoperative evaluation of MSF perfusion, allowing to immediately find and remove ischemic zones, preventing the reconstructive failure. Nowadays, the most used intraoperative technique, beyond the clinical evaluation, to assess MSF perfusion is represented by qualitative indocyanine green (ICG) angiography. ICG is a fluorescence molecule, with a short half-life, that is able to bind plasma proteins, with an excellent security profile and a fast clearance [12, 13]. However, the MSF necrosis rate, even reduced, remains around 10% of cases [11]. To improve this technology, some authors have suggested a quantitative perfusion evaluation, trying to identify a threshold level able to predict skin necrosis, allowing to plan the best reconstructive strategy [14, 15]. On these bases, the main objective of the present study is to find a possible correlation between the MSF perfusion grade, using a qualitative and quantitative assessment, using the SPY® system and the ImageJ® software [16], respectively, and the skin necrosis rate at one month after surgery.

## 2. Materials and Methods

This was a pilot, observational and monocentric study, performed at the EUSOMA-certified Breast Unit ASUGI of Trieste University Hospital. Consecutive patients with breast cancer or BRCA 1-2 genetic mutation scheduled for NAC/skin-sparing or skin-reducing mastectomy between May 2020 and April 2021 were enrolled. Exclusion criteria were age under 18 years old and contraindications to ICG (i.e., allergy to iodine and thyroid disorders).

The main outcome of the study was to find a possible correlation between MSF perfusion grade, using both qualitative and quantitative analysis of ICG fluorescence, and the skin necrosis rate at one month after surgery. The quantitative perfusion measurement was performed for the best vascularized MSF area (ICG-Q_0_) and for the least perfused MSF area (ICG-Q1). Moreover, the relative quantitative perfusion value (ICG-Q%) was calculated comparing the least vascularized area (ICG-Q1) to the best one of the same MSF (ICG-Q_0_).

Necrosis was divided into superficial (SN) and full-thickness (FTN), considering its extension to the subcutaneous tissue [14].

Secondary outcomes were to analyze the following:

- A possible correlation among age, body mass index (BMI), MSF thickness, breast weight, time T0 (interval of time intercurrent between ICG injection and MSF initial perfusion), time Tmax, intercurrent between ICG injection and the highest MSF perfusion grade, time T1 (interval of time intercurrent between ICG injection and the initial perfusion of the least perfused MSF area), ICG-Q_0_, ICG-Q1, and ICG-Q%.
- If age, BMI, MSF thickness, T0, Tmax, T1, ICG-Q_0_, ICG-Q1, and ICG-Q% help in predicting MSF necrosis at one postoperative month.
- If age, BMI, MSF thickness, T0, Tmax, and T1 are useful to predict ICG-Q1 or ICG-Q%.

Moreover, postoperative complications in the presence of seroma, hematoma, SSI, implant removal, and reconstructive failure were evaluated at one month.

### 2.1. Preoperative Evaluation

Patients underwent breast clinical evaluation, mammography and/or ultrasounds, fine needle aspiration cytology (FNAC), and/or tru-cut needle biopsy. According to EUSOMA recommendations, the preoperative workup is comprehensive of a breast magnetic resonance (MR) to evaluate the distance between the tumor and the NAC. The MR was also useful to preoperatively study MSF thickness [17].

Every case has been discussed in a multidisciplinary meeting with all breast specialists (general surgeons, plastic surgeons, oncologists, radiologists, radiotherapists, geneticists, gynecologists, and pathologists) to tailor the treatment for every patient. After that, patients were informed concerning the multidisciplinary discussion and were invited to sign the informed consent to be enrolled in the study.

Before surgery, the following data were collected: age, body mass index (BMI), smoke abuse, arterial hypertension, type II diabetes mellitus, history of previous radiotherapy (RT), chemotherapy (CHT) or breast surgery, ASA (American Society of Anesthesiologists) score, and MSF thickness. Data were prospectively collected in an electronic database.

### 2.2. Surgical Procedure

Every surgery was performed under general anesthesia by an experienced team of general and plastic breast surgeons. Broad-spectrum antibiotic prophylaxis was administered at the induction. Three different mastectomy techniques were adopted: NAC-sparing (NSM), skin-sparing (SSM), and skin-reducing (SRM), together with lymph node biopsy or axillary lymph node dissection, depending on the clinical situation.

For NSM, two kinds of incisions were possible: between the external mammary quadrants or at the lateral third of the submammary fold, according to the tumor position. In the case of submammary incision, a separate axillary incision was performed for the sentinel lymph node biopsy or axillary lymph node dissection when needed.

For the SSM, a lateral-to-medial and periareolar incision was performed. For SRM, a T-inverted incision including NAC was chosen, according to the technique described by Nava et al. [18].

MSF perfusion was evaluated before breast reconstruction. Women were injected intravenously with 0.2 mg/kg of ICG. The interval of time between ICG injection and MSF initial perfusion (T0), the time intercurrent between ICG injection and the higher MSF perfusion grade (Tmax), and the perfusion intensity at Tmax (ICG-Q_0_, ICG-Q1 and ICG-Q%) were recorded.

**Figure 1:**
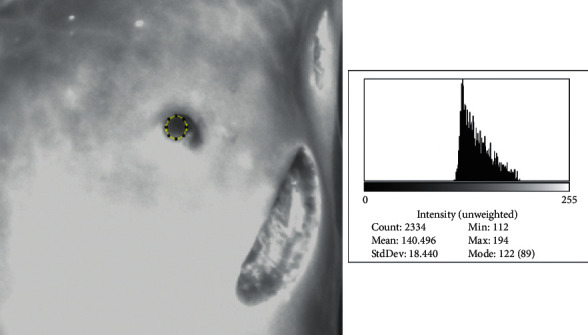
Quantitative perfusion assessment of the less vascularized after a left nipple-sparing mastectomy using ImageJ.

First, a qualitative perfusion assessment was achieved using the SPY Portable Handheld Imager (SPY-PHI®) System (Stryker, Kalamazoo, Michigan, US); after that, the software ImageJ® was used for the quantitative evaluation [16]. From the quantitative analysis, an absolute value of perfusion was measured for the best (ICG-Q_0_) and least (ICG-Q1) vascularized skin area (Figure 1); a relative perfusion value was obtained comparing the absolute perfusion values of the two areas (ICG-Q%). Then, the interval of time between ICG injection and the perfusion of the least vascularized area was recorded (T1).

Therefore, surgery was completed with the reconstructive part, using different strategies, according to patients' characteristics (prepectoral or submuscular implants or tissue expanders). Moreover, breast weight and operative time were collected.

For all patients one or two suction-drains were placed: one in the submuscular pocket and the other in the mastectomy site. In the case of prepectoral breast reconstruction, only one drain had been placed. Concerning the two-stage breast reconstruction, the volume used to fill the expander was based on the thickness and the size of the submuscular pocket. In general, a tissue expander filled with 20% of the total volume was used. The volume of the final implant had not been collected.

### 2.3. Postoperative Care

Venous thrombosis prophylaxis was administered for 15 days or until rehabilitation was completed. Elastic gauzes were used to apply moderate compression for 3 days after surgery, and patients usually wore a specific bra till the third postoperative month. At the one-month follow-up visit, the subsequent data were collected: the presence of seroma, hematoma, SSI, presence of SN or FTN, and reconstructive failure. Patients were divided into two groups: Group 1 in the absence of both SN and FTN and Group 2 in the presence of SN or FTN.

### 2.4. Ethical Aspects

Data were anonymously collected in a protected electronic database for the time needed for their analysis. Hence, they were archived in a private data depository and managed only by the study responsible. The study has been conducted according to Good Clinical Practice, to ethic principles from Helsinki Declaration, and to the current normative on observational studies and approved by the local ethical committee. The informed consent was signed by every patient enrolled.

### 2.5. Statistical Analysis

Nominal and ordinal variables are expressed as number and percentage (arterial hypertension, type II diabetes mellitus, ASA score, smoke abuse, history of RT, CHT or breast surgery, type of surgery performed, position of implant/expander, presence of seroma, hematoma, SSI, SN, FTN, and reconstructive failure); quantitative normal variables as mean ± standard deviation (age, BMI, MSF thickness, T0, T1, ICG-Q_0_, and ICG-Q1); and quantitative nonnormal variables as median and range (breast weight, Tmax, and ICG-Q%). The Shapiro–Wilk test has been used to assess variables' normal distribution. Statistics compared demographic, intra- and postoperative data of Group 1 and Group 2. Normal variables were compared using the Student *T* test; quantitative nonnormal with the Mann–Whitney *U* test; and nominal and ordinal variables with chi-squared and exact Fischer's tests.

A generalized binomial linear model was created to study the effect of age, BMI, breast weight, MSF thickness, T0, Tmax, T1, ICG-Q_0_, ICG-Q1, and ICG-Q% on SN and FTN incidence using the bias reduction in generalized linear models fit method of R software [19]. Moreover, a logarithmic transformation was used to normalize ICG-Q1 and ICG-Q% to create a gaussian generalized linear model to study the effect of age, BMI, breast weight, MSF thickness, T0, Tmax, and T1 on ICG-Q1 and ICG-Q%.

The Pearson correlation test was used to find a possible correlation among normal demographic and intraoperative variables; the Spearman correlation test was used among quantitative nonnormal demographic and intraoperative variables. Confidence intervals of 95% were adopted.

Specificity, sensitivity, and positive and negative predictive values (PPV and NPV) were calculated for relative quantitative perfusion (ICG-Q%) and absolute quantitative perfusion (ICG-Q1).

Furthermore, the receiver operating characteristic (ROC) analysis and the area under the curve (AUC), with 95% confidence interval on the difference between the AUC and 0.5 (Two-tailed test), were calculated for T1. *p* values <0.05 were considered statistically significant. Statistical analysis was conducted using SPSS v23.0 (IBM Corp, Armonk, New York, USA), R v4.0.3 (R Foundation for Statistical Computing) software, and XLSTAT LIFE SCIENCE software (Addinsoft, New York, NY).

## 3. Results

34 women were enrolled in the study from May 2020 to April 2021. All patients matched the inclusion criteria; therefore, none of the 34 was excluded. The two groups do not statistically differ concerning demographic data, except for smoke abuse (*p*=0.008) (Table 1).

**Figure 2:**
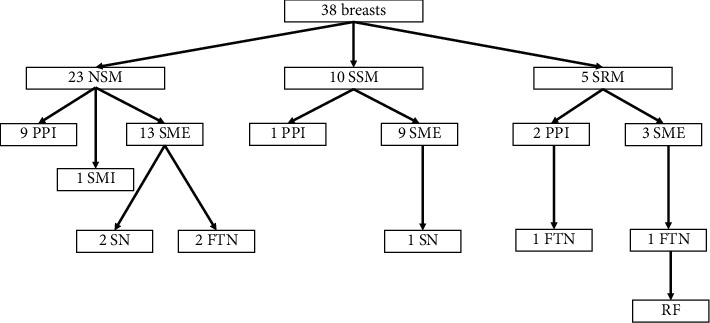
Diagram of the population. Legend: NSM: nipple-sparing mastectomy; SSM: skin-sparing mastectomy; SRM: skin-reducing mastectomy; PPI: prepectoral implant; SMI: submuscular implant; SME: submuscular expander; SN: superficial necrosis; and FTN: full-thickness necrosis.

30 (88.2%) patients underwent unilateral surgery for breast cancer; the others 4 (15.8%) had bilateral surgery for cancer or BRCA1/2 mutation, for a total of 38 breasts. In 23 (60.5%) cases, a NSM was performed; in 10 (26.3%), a SSM was performed; and in 5 (13.2%), a SRM was performed. Concerning patients underwent bilateral surgery, 2 (5.3%) of them had a NSM and 2 (5.3%) a SRM. Breast reconstruction was always immediate: in 12 (31.6%) cases, a prepectoral implant was placed; in one (2.6%) case, a submuscular implant was placed; and in 25 (65.8%), a submuscular expander was placed (Figure 2). FTN was reported in 4 breasts (10.5%); instead, SN in 3 cases (7.9%). Only in one case of FTN (2.6%), surgical debridement and implant removal were needed, with subsequent reconstructive failure. Medical treatment was sufficient in the other cases. No statistically significant difference was found between the two groups for other complications (Table 2).

Concerning intraoperative data, the two groups do not statistically differ for T0, Tmax, ICG-Q1, and ICG-Q0, but they differ for T1 (T1 was longer for Group2 than Group1) and ICG-Q% value (ICG-Q% value was higher for Group1 than Group2) (*p* < 0.05) (Table 2).

The generalized binomial linear model showed that none of the considered factors (age, BMI, breast weight, MSF thickness, T0, Tmax, T1, ICG-Q_0_, ICG-Q1, and ICG-Q%) could predict SN and FTN at one month.

The gaussian generalized linear model to study the effect of age, BMI, breast weight, MSF thickness, T0, Tmax, and T1 on ICG-Q1 and QL% showed that only T1 could statistically predict quantitative absolute and relative perfusion values (*p* < 0.05).

Correlations among demographic and intraoperative variables are reported in Table 3.

A statistically significant positive Pearson correlation was found between

- BMI and age (weak) ⟶ older patients have higher BMI.
- BMI and MSF thickness (weak) ⟶ the higher the BMI is, the thicker MSF are.
- BMI and ICG-Q1 (weak) ⟶ the higher the BMI is, the higher the absolute perfusion value of the least vascularized skin area will be.
- MSF thickness and ICG-Q1 (weak) ⟶ the thicker the MSF are, the higher the absolute perfusion value will be.
- ICG-Q_0_ and ICG-Q1 (weak) ⟶ the higher the absolute perfusion value of the well-vascularized area is, the higher the absolute perfusion value of the least vascularized one will be.

A statistically significant negative Pearson correlation was found between

**Figure 3:**
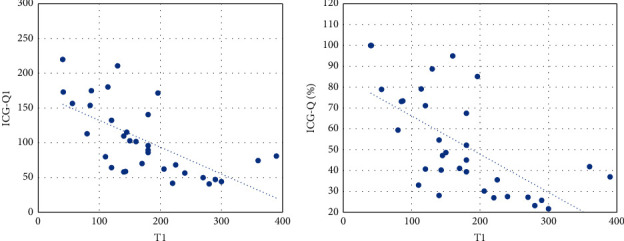
Negative Pearson correlation between T1 and ICG-Q1 (strong correlation) and negative Spearman correlation between ICG-Q% and T1 (strong correlation).

- T1 and ICG-Q1 (strong) ⟶ the longer the time that the ICG needs to reach the least perfused area is, the lower its absolute perfusion value will be (Figure 3).
- ICG-Q1 and ICG-Q% (strong) ⟶ the higher the absolute perfusion value is, the higher the relative one will be.

A statistically significant positive Spearman correlation was found between

- MSF thickness and breast weight (weak) ⟶ the more the breast weights, the thicker the skin flap is.
- T0 and T max (strong) ⟶ the longer the time that ICG needs to start to perfuse the MSF is, the longer the time that it needs to perfuse the MSF at the highest grade is.
- Tmax and T1 (strong) ⟶ the longer Tmax is, the longer the time that the ICG needs to reach the lesser perfuse area will be.

A statistically significant negative Spearman correlation was found between

- Tmax and ICG-Q1 (weak) ⟶ the longer Tmax is, the lower the absolute perfusion value of the least vascularized area will be.
- Tmax and ICG-Q% (weak) ⟶ the longer Tmax is, the lower the relative perfusion value of the least vascularized area will be.
- T1 and ICG-Q% (strong) ⟶ the longer the time between ICG injection and the initial perfusion of the least perfused MSF area is, the lower its relative perfusion value will be (Figure 3).

ICG-Q% and ICG-Q1 were compared in terms of specificity and sensitivity, PPV, and NPV (Table 4). Being the quantitative values nonnormally distributed, we calculated the median relative and absolute values of lesser-perfused skin areas (ICG-Q%: 35.6; ICG-Q1: 68.2) and considered the test positive in case of SN or FTN development. Both values have a sensitivity of 57% and a NPV of 89%; however, the relative value ICG-Q% shows a higher specificity (81% vs 77%), and a higher PPV (40% vs 36%).

**Figure 4:**
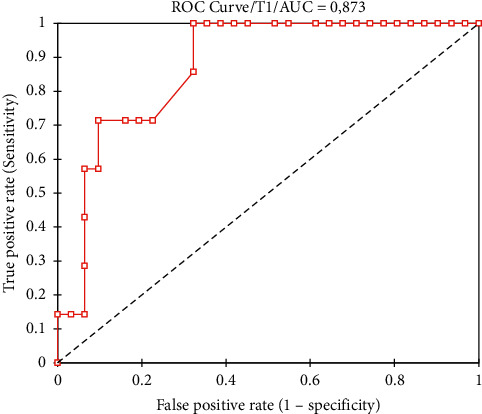
Receiver operating characteristics (ROC) curve of T1.

Moreover, we performed the ROC analysis for T1 (Figure 4). According to our analysis, if T1 is equal to or higher than 170 seconds, there is a high risk of MSF necrosis/epidermolysis, with sensitivity 100%, specificity 68%, PPV 41%, and NPV 100%. The AUC is 0.87 (confidence interval 0.26; 0.49), and it is significantly different from 0.5 (*p* < 0.001).

## 4. Discussion

Breast cancer is the most common type of cancer worldwide, and in the case of surgical treatment, about 25–30% of the women undergo mastectomy [1, 3]. Therefore, postmastectomy breast reconstruction assumed a crucial role in the holistic care of patients affected by breast cancer. Thanks to technological improvement, in the past few years, complex breast reconstruction has become more and more reliable and safe [2, 6, 7, 10, 11].

MSF necrosis is one of the most fearsome complications for IBR, with an incidence of 10–20%, followed by an implant removal in 8–18% of cases, and a delay in systemic therapy beginning, when needed [10, 11].

Historically, MSF intraoperative evaluation was based on flaps color, temperature, capillary refill, and dermal edge bleeding [20]. However, it has been showed to underestimate ischemia and necrosis [10, 14]. In fact, the MSF necrosis rate using only clinical evaluation is around 20% [11], with a necrosis-related reoperation rate of about 10% [10].

Different authors have studied the role of qualitative ICG angiography to reduce the incidence of necrosis in breast reconstruction. ICG is a water-soluble substance, initially developed for infra-red photography, and approved by the Federal Drug Administration in 1959 for use in humans. It is excreted via the liver into the bile upon binding to plasma proteins. It has 3–5 minutes of half-life in humans, allowing its repetitive use during the same surgery and even in the presence of compromised renal function. ICG is detected with near-infrared cameras harboring 806 nm diode-laser, enabling the assessment of blood flow and tissue perfusion in the intraoperative setting [21]. This explains its wide use in different surgical areas [2, 22–25].

Concerning breast reconstruction, one of the most important prerequisites for a successful IBR after NSM, SSM, or SRM is to have well-vascularized skin flaps. ICG angiography plays a pivotal role in this scenario, allowing us to assess skin flaps perfusion before placing the implant, helping for a successful reconstruction [21]. Pruimboom et al. [11] stated that even if with a very low quality of evidence, ICG angiography has a role in reducing MSF necrosis incidence (RR 0.79, 95% CI 0.40 to 1.56; three studies, 573 participants), and other postoperative complications as infection rates (RR 0.91, 95% CI 0.60 to 1.40; four studies, 613 participants), hematoma rates (RR 0.87, 95% CI 0.30 to 2.53; two studies, 459 participants) and seroma rates (RR 1.68, 95% CI 0.41 to 6.80; two studies, 408 participants) compared to the clinical evaluation. They found evidence that qualitative ICG angiography could decrease reoperation rates (RR 0.50, 95% CI 0.35 to 0.72; four studies, 613 participants). Moreover, a review and meta-analysis by Lauritzen and Damsgaard [26] describes how the IBR performed with ICG angiography to evaluate MSF showed a significantly lower risk of major complications, defined as complications requiring surgery/debridement in local or general anesthesia, hematoma, and loss of reconstruction ([OR] = 0.56; 95% CI (0.42–0.76), and *p*=0.0001) as well as a lower risk of reconstruction failure ([OR] = 0.46; 95% CI (0.27–0.82), and *p*=0.0006). The risk of minor complications, defined as ones requiring only conservative treatment, was not significantly reduced ([OR] = 1.05, 95% CI (0.79–1.4), and *p*=0.7). Even if notably reduced, the MSF necrosis rate remains present even when qualitative ICG angiography is adopted.

Therefore, some authors have introduced a quantitative fluorescence evaluation to improve ICG angiography in detecting ischemic areas that might evolve in SN or FTN.

Phillips et al. [14] conducted a prospective clinical trial of tissue expander and implant breast reconstruction with MSF intraoperative evaluation by clinical assessment, ICG angiography, and fluorescein dye angiography. They concluded that ICG angiography is a better predictor of MSF necrosis than fluorescein dye angiography and clinical judgment. However, it overpredicts necrosis in about 72% of cases. For this reason, a quantitative fluorescence analysis is demanded. They performed quantitatively analyzed saved intraoperative videos using SPY-Qs at the 2-minute time point, showing that an absolute perfusion unit less than 3.7 was predictive of postoperative necrosis with 100% sensitivity and 90% specificity.

Newman et al. [15] retrospectively analyzed 20 SPY images from IBR: 10 from breasts that developed flap necrosis and 10 from breasts that underwent adequate healing. Groups were matched for age, BMI, and comorbidities. They evaluated the points of necrosis and points of adequate healing using the SPY-Q postprocessing software, showing a mean relative fluorescence of the necrosis and the adequate healing groups of 25.2% and 43.3%, respectively (*p* < 0.001). The mean absolute fluorescence of the 2 groups was 18.5 and 25.0, respectively (*p*=0.07). They concluded that absolute values may not be sufficiently consistent to establish comparative values across patients, suggesting that quantitative relative perfusion values may increase objectively clinical judgment of flap viability.

Moyer and Losken [27] studied a prospective cohort of one hundred eighteen women who underwent SSM and breast reconstruction. Beyond the intraoperative MSF perfusion evaluation using qualitative ICG angiography, they analyzed the quantitative perfusion value of 15 breasts that developed postoperative skin necrosis. They stated a cutoff perfusion score of 33% below which the surgeon can expect to remove nonviable skin more accurately.

To the best of our knowledge, little is known about the quantitative perfusion assessment of ICG angiography in breast reconstructive surgery. In our case series, we found a statistically significant difference concerning the relative perfusion value between the two groups (52.2%; range 21.7–100 in Group 1 vs 35.6%; range 23.2–67.5 in Group 2; *p*=0.01). Moreover, the time that the least vascularized area requires to be well perfused by ICG (T1) was statistically longer in patients that developed SN or FTN (145.3 ± 72.8 seconds in Group 1 vs 257.9 ± 75.3 seconds in Group 2; *p*=0.001). This means that if a prolonged time T1 and a low relative perfusion value are detected, the probability of MSF necrosis increases, and a reconstructive strategy change could be intraoperatively adopted. In fact, T1 is the only factor that helps in predicting the quantitative absolute and relative perfusion values of the least vascularized area. Moreover, longer are T1, and the time intercurrent between ICG injection and the MSF highest perfusion grade (Tmax), lower are the relative (ICG-Q%) and absolute (ICG-Q1) perfusion values (*p* < 0.05). This implies that the longer is the time that MSF need to be perfused by ICG, the lower the quantitative perfusion value, the higher the possibility of postoperative necrosis will be. Nevertheless, the generalized binomial linear model showed that age, BMI, breast weight, MSF thickness, T0, Tmax, T1, ICG-Q_0_, ICG-Q1, and ICG-Q% are not able to statistically predict SN and FTN. This could be related to the small sample size considered for the study. Moreover, ICG-Q% and ICG-Q1 have both a sensitivity of 57% and an NPV of 89%; however, the relative value ICG-Q% shows a higher specificity (81% vs 77%) and a higher PPV (40% vs 36%). In particular, in our case series, an ICG-Q% value lower than 35.6% was related to an increased incidence of MSF necrosis. The ROC analysis for T1 showed that if T1 is equal to or higher than 170 seconds, there is a high risk of MSF necrosis/epidermolysis, with a sensitivity of 100% and a specificity of 68%. Furthermore, we confirmed that smoke abuse is able to influence the development of FTN and SN at one month (*p* < 0.05) [13, 28].

Our study is limited by the small sample size, the missing final implant volume, and the fact that skin evaluation with ICG angiography was always performed only before placing the implant. The literature does not suggest a clear indication on when to perform ICG angiography. Different authors evaluate the skin perfusion before the implant placement [29, 30]. On the other side, some authors perform ICG angiography after the implant placement with a temporary skin closure [10]. Our approach was related to the fact that in the case of skin hypoperfusion, a reconstructive strategy change could be adopted (for example, submuscular implant placement instead of prepectoral) and the skin could be excised before the implant placement.

Advantages are the prospective data collection, and all surgery was performed by an experienced breast-surgeon team.

## 5. Conclusions

ICG angiography is a valuable method to assess MSF perfusion during surgery. However, even reducing the necrosis rate does not eliminate it. For this reason, the qualitative evaluation should be combined with a quantitative analysis. Moreover, the T1 may be useful to help predict necrosis. Considering these factors, an intraoperatively change in reconstructive strategy could be adopted, preventing reconstructive failure. However, other studies are needed to confirm our data.

## Figures and Tables

**Table 1:** Demographic data of the population studied.

	Group 1–27 women	Group 2 (SN + FTN)–7 women	*p* value
Age	49.9 ± 11.6	53.7 ± 10.9	0.4
BMI	22.1 ± 2.4	21.7 ± 3.4	0.7
AH	2 (7.4)	1 (14.3)	0.5
DM II	2 (7.4)	0	1.0
ASA score			1.0
I	22 (81.5)	6 (85.7)	
II	5 (18.5)	1 (14.3)	
Smokers			0.008
Current	1 (3.7)	3 (42.9)	
Ex	2 (7.4)	0	

	Group 1–31 breasts	Group 2 (SN + FTN)-breasts	

History of			
RT	1 (3.7)	1 (14.3)	0.3
CHT	3 (11.1)	3 (42.9)	0.6
Breast surgery	5 (18.5)	1 (14.3)	1.0
BW (g)	199 (60–500)	405 (180–612)	0.3
MSFT (mm)	8.7 ± 2.3	8.8 ± 3.3	0.9
Type of surgery			0.36
NSM	19 (61.3)	4 (57.1)	
SSM	9 (29.0)	1 (14.3)	
RSM	3 (9.7)	2 (28.6)	
Position of implant/expander			0.4
Prepectoral	11 (35.5)	1 (14.3)	
Submuscular	20 (64.5)	6 (85.7)	

Nominal and ordinal variables are expressed as number and percentages; normal variables as mean ± standard deviation; quantitative nonnormal variables as median and range. BMI: body mass index; AH: arterial hypertension; RT: radiotherapy; CHT: chemotherapy; DM II: type II diabetes mellitus; BW: breast weight; MSFT: mastectomy skin flaps thickness; NSM: nipple-sparing mastectomy; SSM: skin-sparing mastectomy; and SRM: skin-reducing mastectomy.

**Table 2:** Intraoperative data and other postoperative complications.

	Group 1–31 breasts	Group 2 (SN + FTN)–7 breasts	*p* value
*Intraoperative data*			
Operative time (sec)	190 (130–400)	201.5 (158–400)	0.8
T0 (sec)	37.6 ± 19.2	44.9 ± 16.7	0.4
T max (sec)	80 (38–167)	110 (65–150)	0.2
T1 (sec)	145.3 ± 72.8	257.9 ± 75.3	0.001
ICG-Q_0_	198.2 ± 31.6	190.2 ± 17.5	0.5
ICG-Q1	112.7 ± 49.7	71.0 ± 33.8	0.08
ICG-Q%	52.2 (21.7–100)	35.6 (23.2–67.5)	0.01

*Other postoperative complications*			
Seroma	2 (6.5)	1 (14.3)	0.1
Hematoma	2 (6.5)	1 (14.3)	
SSI	0	0	
Reconstructive failure	0	1 (14.3)	

Nominal variables are expressed as number and percentages; normal variables as mean ± standard deviation; quantitative nonnormal variables as median and range. SN: superficial necrosis; FTN: full-thickness necrosis; ICG-Q_0_: quantitative absolute perfusion value of the best MSF vascularized area; ICG-Q1: quantitative absolute perfusion value of the least MSF vascularized area; and ICG-Q%: quantitative relative perfusion value of the least MSF vascularized area.

**Table 3:** Pearson and Spearman correlation among demographic and intraoperative variables.

*Pearson correlation among demographic and intraoperative variables*										
	Age	BMI	MSFT	T0	T1		ICG-Q_0_		ICG-Q1	
Age	**1**	**0.414**	0.030	0.051	0.002		0.001		0.182	
BMI	**0.414**	**1**	**0.555**	0.104	−0.033		0.176		**0.346**	
SFT	0.030	**0.555**	**1**	0.036	0.002		0.276		**0.359**	
T0	0.051	0.104	0.036	**1**	0.238		0.310		0.046	
T1	0.002	−0.033	0.002	0.238	**1**		−0.143		**−0.643**	
ICG-Q_0_	0.001	0.176	0.276	0.310	−0.143		**1**		**0.409**	
ICG-Q1	0.182	**0.346**	**0.359**	0.046	**−0.643**		**0.409**		**1**	

*Spearman correlation among demographic and intraoperative variables*										
	Age	BMI	MSFT	BW	T0	Tmax	T1	ICG-Q_0_	ICG-Q1	ICG-Q%

BW	−0.155	0.252	**0.566**	**1**	−0.146	0.025	0.250	−0.124	−0.091	−0.043
*T* max	−0.071	−0.082	−0.011	0.025	**0.623**	**1**	**0.614**	0.042	**−0.432**	**−0.444**
ICG-Q%	0.103	0.287	0.252	−0.043	−0.071	**−0.444**	**−0.688**	0.178	**0.940**	**1**

Values in bold are different from 0 with a significance level alpha = 0.05. BMI: body mass index; BW: breast weight; MSFT: mastectomy skin flaps thickness; ICG-Q_0_: quantitative absolute perfusion value of the best MSF vascularized area; ICG-Q1: quantitative absolute perfusion value of the least MSF vascularized area; and ICG-Q%: quantitative relative perfusion value of the least MSF vascularized area.

**Table 4:** Sensitivity, specificity, and positive and negative predictive values (PPV and NPV) for relative quantitative perfusion (ICG-Q%) and absolute quantitative perfusion (ICG-Q1) of the least vascularized MSF areas.

	ICG-Q1 (%)	ICG-Q%
Sensitivity	57	57
Specificity	77	81
PPV	36	40
NPV	89	89

## Data Availability

The data that support the findings of this study are available from the corresponding author upon reasonable request.
